# Study on the Effect of *Mentha × piperita* L. Essential Oil on Electroencephalography upon Stimulation with Different Visual Effects

**DOI:** 10.3390/molecules27134059

**Published:** 2022-06-24

**Authors:** Shifan Lin, Yue Wang, Kaiwen Wu, Genfa Yu, Chuanxiang Liu, Chang Su, Fengping Yi

**Affiliations:** 1 Department of Perfume and Aroma Technology, Shanghai Institute of Technology, Shanghai 201418, China; linshifan@yeah.net (S.L.); arabella623@icloud.com (Y.W.); kevien9808@gmail.com (K.W.); hedioneyu@163.com (G.Y.); suchang@sit.edu.cn (C.S.); 2 School of Chemical and Environmental Engineering, Shanghai Institute of Technology, Shanghai 201418, China; cxliu@sit.edu.cn

**Keywords:** peppermint essential oil, colour, inhalation, electroencephalograph

## Abstract

Essential oils have long been used to fight infections and treat various diseases. Peppermint (*Mentha × piperita* L.) is an herbal medicine that has been widely used in daily life since ancient times, and it has a wide range of applications in food, cosmetics, and medicine. Mint oil is refreshing because of its cool and comfortable smell; therefore, it is often used in ethnopharmacological studies. The present study investigated the effects of peppermint essential oil in electroencephalographic activity response to various visual stimuli. The electroencephalographic changes of participants during peppermint essential oil inhalation under white, red, and blue colour stimulations were recorded. A rapid Fourier transform analysis was used to examine the electroencephalograph power spectra of the various microstates induced by inhaling the oils. Peppermint essential oil had various effects on the brain when subjected to different visual stimuli. Alpha waves increased in the prefrontal area in the white-sniffing group, which facilitated learning and thinking. In the blue-sniffing group, the changes were less pronounced than those in the red group, and the increased alpha wave activity in the occipital area was more controlled, indicating that the participants’ visual function increased in this state. Based on EEG investigations, this is the first study to indicate that vision influences the effects of peppermint essential oils. Hence, the results of this study support the use of essential oils in a broader context to serve as a resource for future studies on the effects of different types of essential oils.

## 1. Introduction

Essential oils (EOs), consisting of volatile compounds, have various biological characteristics, such as antioxidant, antibacterial, and anticancer properties [1]. They are part of traditional medicines, such as the traditional Chinese medicine Ayurveda, as well as modern herbal and aromatherapies. They were probably used as early as 5000 BCE in ancient Egypt [2].

Aromatherapy is derived from the psychological effects of the odours of EOs and the physiological effects of inhaled volatile compounds. In chronic hemodialysis patients, inhaling Hiba oil is an effective, noninvasive means for the treatment of depression and anxiety, and Lavender alleviates anxiety [3]. Since ancient times, EOs have been thought to regulate mood, and previous research has reported that the smell of EOs from Lavandula and Mentha (the plant name has been checked with http://www.theplantlist.org, accessed on 25 April 2022) has a calming effect. EOs have also been used in the clinical treatment of patients and as interventions for treating depression, reducing insomnia, and relieving pain [4].

Peppermint, which belongs to the Lamiaceae family, is a common herb used in food and nonfood applications. It has a cooling, sweet, and herbaceous smell that is appreciated by consumers. Peppermint essential oil (PEO), which can be obtained via hydrodistillation, is an oily liquid consisting of volatile compounds. The main volatile compounds of PEO are menthol and menthone, of which the characterising compound is menthol furan. Previous reports have found that these compounds are the main aroma-imparting compounds of PEO with low threshold values. Peppermint plants and their EOs have traditionally been used to repel insects and treat nausea, headaches, and various skin conditions [5]. PEO has also been used as an aromatic product to keep people awake. The results of animal experiments also indicate anxiety and vigilance effects in animals, which are caused by the analgesic and anaesthetic effects of PEO on the central nervous system [6]. When used as a clinical treatment, PEO greatly reduces fatigue and relieves pain in patients [7,8]; thus, PEO is widely used in both daily life and assisted medicine treatment.

Current studies on brain electrophysiology suggest that aroma can affect brain activity and cognitive function, which can be evaluated via electroencephalography (EEG) and event-related potentials (ERPs) [9,10]. Aromatic compounds in EOs can affect the nervous system via smell. The aroma of EOs activates synaptic transmission to the central nervous system, and the signal input eventually rises to several brain areas responsible for olfactory perception, autonomic balance, and other advanced brain functions, thereby affecting the body and the mind [11,12]. Most past EEG studies of odour effects have shown that alpha activity can be increased by taking several EOs, such as lavender, sandalwood, and chamomile oils. Diego et al. [1] found electroencephalogram (EEG) readings to show increased beta-power following lavender inhalation, implying neurological sedation, and corroborating subjective reports of calmness, while jasmine has been demonstrated to produce increased alpha-power in the frontal cortices, indicative of increased arousal [13]. However, few studies have examined the effects of EOs on brain waves in different test environments. The central nervous system is also affected by visual stimulation; therefore, EOs may have different effects in different visual environments. Physiological changes induced by aroma exposure may be related to the regulatory activity of the optic nervous system. Studies have shown that the colour of office partitions have different effects on a person’s mood. The results show that blue partitions can reduce fatigue and increase the autonomous response [14].

Thus, EEG could be used as a neurophysiological assessment tool to study the changes in the physiological activities of the brain, reflecting the brain state or brain function, which can be measured through recording or imaging. The frequency of brain waves is between 0.05–500 Hz, comprising the delta waves (0–4 Hz), theta waves (4–8 Hz), alpha waves (8–13 Hz), and beta waves (13–30 Hz), among others. Different brain waves reflect various effects on the brain [15,16].

As the potential use of PEO products in daily life has not been analysed, the purpose of this study was to evaluate the effects of PEO on human cognition under different visual stimuli. It was believed that colour and olfaction could have a synergistic effect. The composition of PEO was assessed via Gas-Chromatography Mass Spectrometry (GC-MS), and EEG was used to record changes in the brain waves. This study used PEO to investigate the synergistic influence of scent and vision and introduced a new approach to studying the effects of EOs and provided support for naturopathy and complementary medicine.

## 2. Results

### 2.1. Volatile Chemicals Comprising PEO

According to GC-MS results, substances with matching values less than 70 were filtered, and the retention indexes were calculated based on the n-alkanes using the Kovats index. The olfactory components of PEO were identified in Table 1, with a total of 59 compounds, including terpenes (14), alcohols (11), aldehydes (4), ketones (10), acids (2), esters (7), phenols (2), and other organic compounds (9). Alcohols and terpenes account for 49.82 percent of the total content.

### 2.2. Comparison of Amplitudes before and after Inhalation of PEO

The spectrograms and EEG data of the subjects at the time of the experiment were collected by running an FFT on the clean EEG using MATLAB after preprocessing the EEG.

#### 2.2.1. Comparison of Spectrograms

**Figure 1:**
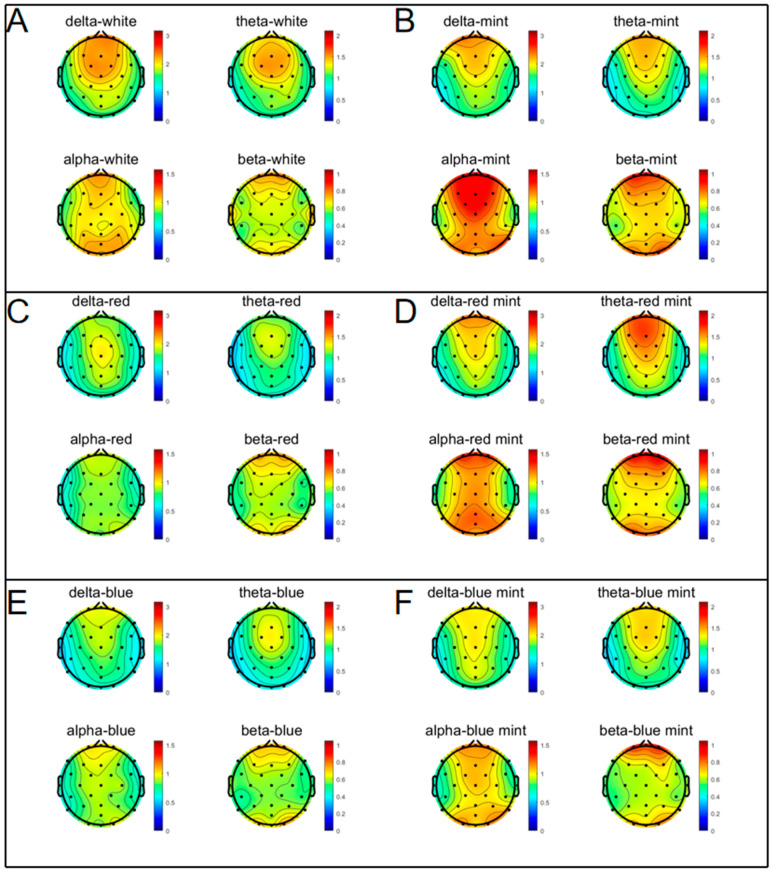
EEG spectral spectrograms before and after inhalation of EOs. Using FFT in EEGLAB software, the brain activity of 20 subjects in the same microstate was summarised as a spectrogram. (**A**) White without PEO. (**B**) White with PEO. (**C**) Red without PEO. (**D**) Red with PEO. (**E**) Blue without PEO. (**F**) Blue with PEO. Significant changes occurred in each region during the inhalation of EOs.

The EEG spectrogram vividly illustrates the subjects’ brain activity in different microstates before and after inhalation of PEO. The delta and theta waves were mainly found in the Fp and F regions. Beta waves were primarily found in the Fp and O areas, whereas alpha waves were found all throughout the brain. Theta waves in the white group tended to diminish slightly during PEO inhalation. Delta, theta, alpha, and beta increased to varying degrees of amplitude in all other groups, as shown in Figure 1.

#### 2.2.2. Variations in the Ratio of Each Electrode Point

The electrode caps defined the electrode groups responsible for each brain region. The electrodes were used to study the activity from each brain region specifically. Based on the distribution of delta, theta, alpha, and beta in the spectrogram, the Fp, F, P, and O areas of the brain were also chosen for significance analysis.

**Figure 2:**
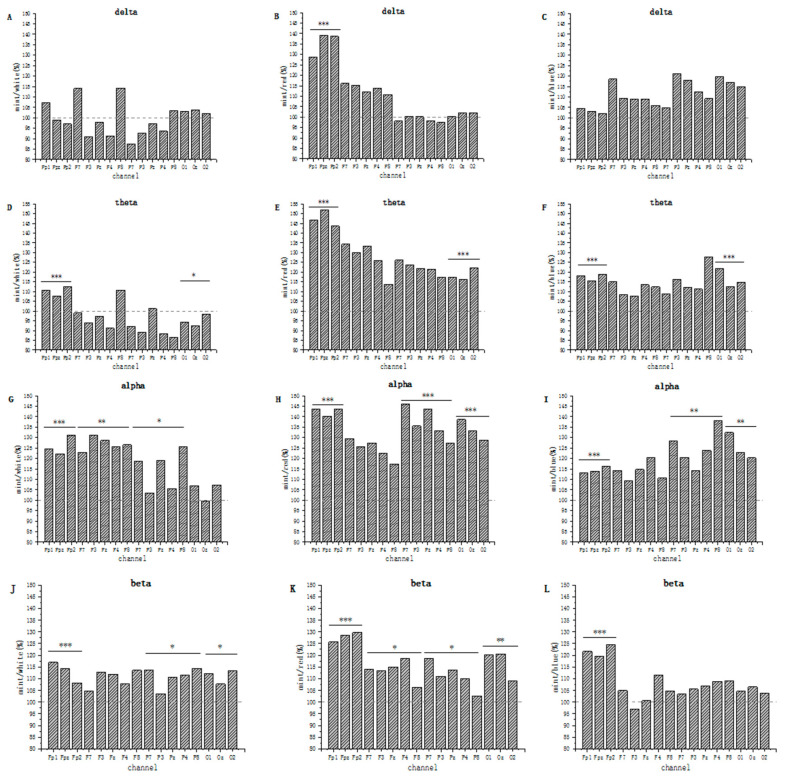
Single-channel analysis comparison graph for each group, comparing bands of the same channel in the same colour with or without PEO inhalation. (**A**,**D**,**G**,**J**) are white groups, (**B**,**E**,**H**,**K**) are red groups, and (**C**,**F**,**I**,**L**) are blue groups (*p*-values: * < 0.05, ** < 0.001, *** < 0.0001. Relevant data can be found in supplementary materials.).

A comparison graph for the PEO/no PEO groups was created using a single-channel analysis in Figure 2. Delta waves were mainly distributed in the forebrain area in the white sniffing and inhaling groups, with an overall decrease in energy but no significant change, as shown in Figure 2A. Theta waves were mostly found in the forebrain, with a distinct prevalence in the Fp area, which amplitude was increased by approximately 10% compared with the control group, as shown in Figure 2D. In the PEO group, alpha wave activity was more pronounced, which was focused on the Fp and F regions. The distribution of alpha waves from the left to the right brain tended to shift, with the prevalence in the Fp2 and F3 regions increasing by 31%, as shown in Figure 2G. In this group, the beta distribution grew throughout the brain, as shown in Figure 2J.

PEO inhalation also caused a substantial increase in the red background in Figure 2B,E,H,K. At this time, the PEO group showed significant increases in delta, theta, alpha, and beta wave activity in the Fp region of up to 51%. Theta, alpha, and beta wave activity increased significantly in region O, with alpha wave activity reaching 30% on average, and were distributed to the left side of the brain.

The effect of PEO on EEG was less significant in the blue background group than in the red background group in Figure 2C,F,I,L. In the PEO group, the increase in theta waves was most significant in areas Fp and O when subjects inhaled PEO. The largest shift in alpha wave activity was observed throughout the brain, specifically in the Fp, P, and O regions, with a 30% increase in the O1 region in Figure 2I. Beta wave activity was specifically centred in the Fp region in Figure 2L.

### 2.3. Comparison of Energies before and after PEO Inhalation

#### 2.3.1. Results of Energy Comparisons

According to the results of the functional brain area energy shown in Table 2, the white and red sniffing and inhalation groups showed significant changes in the Fp area after PEO inhalation. The alpha and delta waves in the blue group also changed significantly. The alpha waves in the white group and theta and beta waves in the red group both shifted to the F region. The alpha waves in the P region changed to varying extents. In the O area, the red group experienced large changes in theta, alpha, and beta wave activity. In contrast, the blue group experienced major changes only in delta and theta wave activity in this region.

#### 2.3.2. Energy Changes in Alpha, Beta Waves

**Figure 3:**
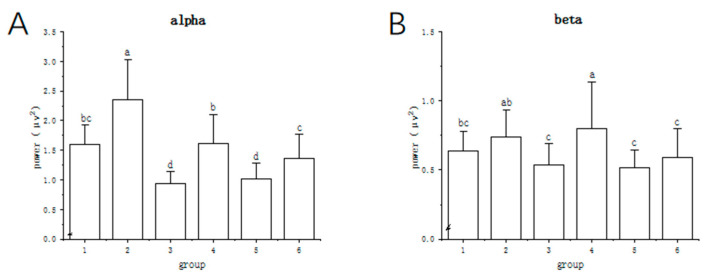
Alpha and beta wave activity under six states. The six different states, defined as microstates 1 to 6, are white, white with PEO, red, red with PEO, blue, and blue with PEO, respectively. (**A**) Significant differences in alpha band. (**B**) Significant differences in beta band. In the same group, values followed by different letters were significantly different (*p* < 0.05).

Six different datasets were established to evaluate the effects of PEO inhalation on the brain when stimulated using different colours. The mean patterns of the alpha and beta waves during PEO inhalation were subjected to ANOVA univariate analysis using various datasets in Figure 3A,B. The inclusion of PEO resulted in a considerable increase in alpha wave activity. Moreover, the beta waves shifted dramatically in the red group.

## 3. Discussion

Out of the more than 20 active components derived from EOs, 2/3 of the alcohols and terpenes recovered had anxiolytic effects on rodents [17]. Similarly, terpenoids and alkanes, the volatile components of camphor, have been demonstrated to have a favourable adjuvant effect on hypertension in elderly human patients [18]. Alcohols and terpenoids are the primary components found in PEO that we use, and the anxiolytic and refreshing properties of PEO have been well-established.

Both alpha and beta wave activity increased after the inhalation of PEO. The increase in alpha wave power can be observed in several EOs, such as white Champaka, Eucalyptus, Lavender, and Zizyphus jujuba oils [19,20,21]. This alteration is related to psychometric responses, a relaxing effect, an increase in attention, and relaxation [22]. PEO has a stress-relieving and antianxiety effect, as shown by the general shift in alpha wave activity. The presence of beta waves in the red state implies that the subjects were more alert, while PEO inhalation increased the subjects’ focus.

According to our findings, the effects of EO inhalation on different parts of the brain are statistically significant but also have some practical implications. Each part of the brain is responsible for separate functions. Mental functions are denoted by Fp, thinking functions by F, somatosensory functions by p, and optical functions by O. The left and right sides of the brain have different divisions of labour, with the left brain being responsible for logical analysis and the right brain for artistic appreciation [23].

Undiluted citrus EOs were able to decrease the power of alpha waves while increasing those in beta waves, indicating that it has a stimulatory effect [24]. However, when the subjects were exposed to the same scent for an extended time, the olfactory receptors create a response that weakens over time and becomes more suited to the environment [25,26]. Spectrograms were used to show the differences in brain activity between the PEO and non-PEO groups. We have shown that combining amplitude analysis on a single channel with amplitude analysis on many channels is feasible. With this, the changes in EEG can be displayed clearly.

When the subjects were presented with a white picture, they were emotionally stable following the PEO effect. The Fp region, which indicates the mental function, showed an increase in the activity of the four waves, so attention was more concentrated than in the blank group. The F and P portions of the frontal lobes of the brain showed a considerable change in alpha wave activity. The subjects’ mental and cognitive states were awake and calm; they had the ability to focus on a specific event or task, unaffected by extraneous stimuli, and the brain was not easily fatigued. At this time, the theta waves in O were diminished, and visual function was not activated. Hence, PEO inhalation can renew the brain, stimulate the soul, and enhance work efficiency in a neutral environment.

Long periods of exposure to red-coloured environments can produce anxiety and unstable emotions. This can make people feel that time is passing more quickly, which is why red is frequently chosen as the main colour in fast food restaurant decors [12]. There was a large increase in delta and theta wave activity in the forebrain area after adding PEO when the subjects were placed in the red state. Moreover, the subjects experienced slow brain activity, which had a significant relaxation effect. There was a considerable increase in alpha and beta waves and active brain activity among the subjects. However, excessive levels of beta waves can also cause anxiety [27,28]. It can be seen that PEO may increase the discomfort and tension created by a warm environment.

Blue has the opposite effect on people as warm colours do. Most blue goods are employed in hospitals because cool environments can provide a sense of tranquillity and calm. By adding PEO to the visual stimulation caused by the blue colour, the increase in delta wave activity in the hindbrain resulted in a feeling of physical relaxation in the subjects. The theta and alpha wave values also increased in the Fp and O regions. The subjects showed improved visual discrimination and were in a deep state of mental relaxation; however, untrained people may have slept during this period. Beta wave activity increased slightly in the Fp area; the presence of these waves aid in improving communication and planning. In a cool environment, PEO induces a sleepy state and relaxing effect on the body, but it has no discernible effect on increasing concentration.

The use of EEG in this experiment increases the scientificity of the results. However, the method is too single, and there are many factors that affect the effect of EO. Based on this experiment, we found that vision can affect the role of smell. In future research, we can try to use colour with EO to improve the effect of plant aromatherapy. Physiological detection and subjective emotion analysis can be added into the analysis method to enrich and complete the analysis results.

## 4. Materials and Methods

### 4.1. GC-MS Analysis of PEO

PEO was purchased from Beijing Zoteq Co., Ltd. (Beijing, China) and stored at 4 °C until use. A sample was kept in the Perfume Laboratory in the Department of Perfume and Aroma, Shanghai Institute of Technology.

GC-MS analysis of PEO was carried out using an Agilent GC-6890 coupled with MS-5973 (Santa Clara, CA, USA). Separation was performed on an HP-Innowax column (60 m × 0.25 mm × 0.25 μm; Agilent, California, USA). GC was operated under temperature-programmed conditions from 40 °C to 230 °C at 3 °C/min and held for 5 min. The samples were analysed in an undivided mode. Helium was used as the carrier gas at a flow rate of 0.8 mL/min and the MS ionisation mode used was electron impact (70 eV).

The retention indexes of each composition relative to n-alkanes (C8–24) under the same operating conditions were calculated using Kovats indexes.

### 4.2. Preparation of Colours

Colours can be classified as cool, warm, or neutral according to the division of a spatial colour design. Blue dominates the cold colours, red dominates the warm colours, while white dominates the neutral colours. In one study [29,30], red and green were the most exciting hues, whereas blue and yellow were the least attractive.

Red (255, 0, 0), blue (0, 0, 255), and white (255, 255, 255) in the standard colour space were used in this experiment to improve the subjects’ colour perception. Each colour image was rectangular (400 × 400 pt, 72 ppi) and was presented in the centre of a computer screen against a white background with no colour names. The colours of the computer monitor were adjusted to present the most accurate colours created with Adobe Illustrator (AI) drawing software.

### 4.3. Subjects

Twenty healthy, right-handed participants (n = 20; F = 10, M = 10, age range 20–30 years) were selected to participate in this study. All subjects claimed to have no problems with smell, vision, or substance abuse and that they had had at least 8 h of sleep before the test. They had no intake of alcoholic or caffeinated beverages within 24 h and did not take any psychotropic drugs within 7 days. This study followed the guidelines of the Declaration of Helsinki and Tokyo for humans and was approved by the Ethics Committee of Shanghai Jiao Tong University with approval #B2021153I, and informed consent was obtained.

### 4.4. Experimental Design

The experiment was conducted in a fixed place, which was equipped with a fresh air system and air conditioning, far from the interference of humans for one month. The temperature of the place was precisely controlled at 25 °C, the humidity was 70%, and the noise level was maintained at less than 40 dB. Before analysis, the subjects washed their hair with an unscented shampoo and their hair was blow-dried. The subjects wore the electrode cap appropriately before injecting a conductive adhesive into the electrode channel to reduce the resistance to less than 5 kω.

**Figure 4:**
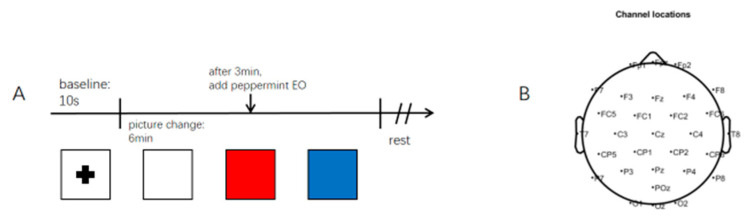
Experimental flow chart ((**A**) Experimental process. (**B**) Potential distribution).

Approximately 0.10 g PEO was added to scented wood. At the beginning of the experiment, the scented wood was placed 7–9 cm below the nose of the subjects to ensure that the aroma was smelled. Sun and Moon [31] used positive and negative images to induce emotions in the volunteers. In this experiment, red and blue pictures were used to make volunteers feel a change in their environment. Participants were also asked to sit in a comfortable chair and concentrate on the blank recording (white, without EO), visual stimuli (red and blue without EO), and visual and olfactory stimuli (white, red, and blue, with EO) for three minutes in Figure 4A.

### 4.5. EEG Signal Acquisition

EEG recordings (eggoTM mylab, ANT Neuro, Hengelo, Netherlands) were recorded using a 32-electrode system. These recordings consist of the prefrontal (Fp), frontal (F), central (C), parietal (P), and occipital (O) regions. Figure 4B is the distribution of electrodes, with reference electrodes M1 and M2 removed. The EEG sampling rate of the measured subjects was 500 Hz, and the data were filtered in the range of 1–40 Hz.

The value of each signal was determined using the sampling rate and duration of time. Owing to the periodicity of the signal at the electrode sites from 32 channels and 20 subjects, the signals were segmented into 2 s time intervals at a sampling rate of 500 Hz during the test, with a segment length L = 500 × 2 s. All time points from the original EEG signal were subjected to a Fourier transform (FFT). Delta, theta, alpha, and beta waves were extracted from four channels [32].

The strength of electrical wave activity within the brain can be described using spectrograms and single-channel analyses. The energy of brain waves was utilised to quantify the results, as well as to synthesise the alpha and beta wave data of the subjects in the same state [33,34]. Hence, it is crucial to determine whether EOs affect the human brain, as this could change volunteers’ moods.

### 4.6. Statistical Analysis

All data sets that passed the normality test were analysed using one-way analysis of variance (ANOVA) to determine the effect of the various treatment factors. Multiple comparisons were performed using the least significant difference (LSD) method to determine statistically significant differences (*p* < 0.05).

## 5. Conclusions

The effect of PEO was substantially better in neutral and warm environments than in cool environments. PEO inhalation invigorated the mind, improved concentration, and facilitated learning and thinking in neutral circumstances, where alpha wave activity increased by up to 31%. The subjects were less stable in warm situations, where beta wave activity was the highest. PEO showed obvious visual and physical relaxation impacts with beneficial results. Therefore, the effects of the same EO may differ in different visual environments.

## Figures and Tables

**Table 1:** Components of peppermint oil as determined via GC-MS.

NO.	RI ^a^	RI ^b^	Compound ^c^	Area% ^d^
			Terpenes	
1	1016	1022	**(+)-Limonene**	15.99%
2	1118	1116	β-pinene	7.71%
3	924	994	pinene	4.88%
4	1032	1097	α-Pinene	1.29%
6	1255	1251	γ-Terpinene	0.39%
7	1695	1632	(+)-ledene	0.23%
8	1662	1619	α-Caryophyllene	0.17%
9	1755	1673	(+)-δ-cadinene	0.15%
10	1290	1322	Terpinolene	0.15%
11	1458	1316	ε-Muurolene	0.14%
12	1130	1086	Sabinene	0.04%
13	1878	1772	1-Methylnaphthalene	0.03%
14	899	894	fenchene	0.02%
			Alcohols	
15	1172	1277	**L-Menthol**	11.57%
16	1380	1381	3-Octanol	3.78%
17	1614	1597	Menthol	1.91%
18	1710	1725	α-terpineol	0.95%
19	1363	1358	cis-3-Hexen-1-ol	0.10%
20	1642	1629	Isoborneol	0.07%
21	1764	1766	Citronellol	0.07%
22	1872	1882	Phenylethyl alcohol	0.07%
23	1820	1820	2-(4-Methylphenyl)propan-2-ol	0.04%
24	1890	1854	Benzyl alcohol	0.03%
25	2138	2159	Spathulenol	0.03%
			Aldehydes	
26	960	1160	Benzaldehyde	0.07%
27	2055	2085	4-Methoxybenzaldehyde	0.03%
28	2115	2088	3-Formylbenzoic acid	0.01%
29	2341	1777	Isophthalaldehyde	0.01%
			Ketones	
30	1380	1450	**l-menthone**	20.14%
31	1130	1140	p-Menthone	6.43%
32	1740	1761	piperitone	2.95%
33	1662	1607	Pulegone	1.84%
34	975	1010	(3R)-3-Methylcyclohexan-1-one	0.57%
35	937	1015	3-Methylcyclohexanone	0.37%
36	1234	1362	(+)-carvone	0.34%
37	1940	1915	Jasmone	0.03%
38	1832	1861	3,4-Dimethylacetophenone	0.02%
39	1867	1837	Geranylacetone	0.01%
			Acids	
40	2448	2515	benzoic acid	0.02%
41	2187	2060	4-Ethylbenzoic acid	0.01%
			Esters	
42	1594	1543	Isomenthol acetate	8.58%
43	1574	1548	(−)-Menthyl Acetate	3.67%
44	1770	1786	Methyl salicylate	0.11%
45	1314	1329	menthalactone	0.19%
46	2655	2584	benzyl benzoate	0.03%
47	2014	1920	Dibutyl phthalate	0.01%
48	2218	2234	Methyl palmitate	0.01%
			Phenols	
49	1265	1298	Thymol	0.03%
50	2156	2191	Eugenol	0.01%
			Other	
51	932	989	2,6-Dimethyloctane	0.01%
52	1279	1318	cis-Anethol	0.10%
53	1029	929	o-Cymene	0.67%
54	1414	1412	2-p-Tolyl-1-propene	0.33%
55	1152	1204	5-tert-Butyl-m-xylene	0.01%
56	1497	1197	menthofuran	2.16%
57	1056	969	m-cymene	1.36%
58	1400	1465	2,6-dimethylnaphthalene	0.03%
59	1989	1953	Caryophyllene Oxide	0.03%

^a^ Retention index calculated from the Kovats index. ^b^ Retention index for the chemical composition from the NIST library. ^c^ Components identified in the highest yield are shown in bold. ^d^ Percentage of peak area of each component to the total peak area.

**Table 2:** Results of the significance analysis of PEO on the Fp, F, P, and O regions under stimulation with different colours.

Site	Colour	Wave	Before Inhalation (μv^2^)	After Inhalation (μv^2^)
Fp	white	delta	8.69 ± 0.25	10.26 ± 0.82 **
		theta	2.72 ± 0.29	4.05 ± 0.13 **
		alpha	1.94 ± 0.16	3.41 ± 0.04 ***
		beta	0.89 ± 0.02	1.17 ± 0.10 **
	red	delta	4.60 ± 0.45	7.90 ± 0.34 ***
		theta	1.76 ± 0.03	7.19 ± 0.96 ***
		alpha	1.18 ± 0.05	2.39 ± 0.14 ***
		beta	0.88 ± 0.09	1.68 ± 0.12 ***
	blue	delta	6.07 ± 0.26	6.18 ± 0.45
		theta	1.95 ± 0.04	2.68 ± 0.13
		alpha	1.45 ± 0.05	1.98 ± 0.05 ***
		beta	0.46 ± 0.03	1.13 ± 0.08 **
F	white	delta	5.77 ± 1.30	7.35 ± 1.68
		theta	2.43 ± 0.76	2.32 ± 0.57
		alpha	1.36 ± 0.35	2.47 ± 0.61 ***
		beta	0.59 ± 0.10	0.74 ± 0.16
	red	delta	3.60 ± 0.98	4.27 ± 1.05
		theta	1.56 ± 0.57	4.64 ± 2.84 **
		alpha	0.88 ± 0.22	1.36 ± 0.39
		beta	0.54 ± 0.07	0.82 ± 0.18 **
	blue	delta	3.88 ± 0.89	4.65 ± 1.00
		theta	1.64 ± 0.64	1.88 ± 0.59
		alpha	0.92 ± 0.22	1.25 ± 0.30
		beta	0.52 ± 0.09	0.54 ± 0.10
P	white	delta	3.77 ± 0.85	3.59 ± 1.06
		theta	1.64 ± 0.28	1.28 ± 0.39
		alpha	1.56 ± 0.24	2.26 ± 0.32 ***
		beta	0.54 ± 0.05	0.65 ± 0.06 *
	red	delta	3.32 ± 1.02	3.29 ± 1.14
		theta	1.09 ± 0.32	1.64 ± 0.43 *
		alpha	0.92 ± 0.16	1.76 ± 0.10 ***
		beta	0.47 ± 0.05	0.62 ± 0.04 ***
	blue	delta	2.71 ± 0.70	3.61 ± 1.15
		theta	1.08 ± 0.35	1.44 ± 0.42
		alpha	0.92 ± 0.17	1.43 ± 0.35 **
		beta	0.46 ± 0.06	0.52 ± 0.08
O	white	delta	3.28 ± 0.23	3.42 ± 0.05
		theta	1.47 ± 0.14	1.09 ± 0.06 ***
		alpha	1.99 ± 0.06	2.27 ± 0.36
		beta	0.78 ± 0.04	0.94 ± 0.10
	red	delta	2.82 ± 0.03	2.88 ± 0.19
		theta	1.01 ± 0.04	1.48 ± 0.04 ***
		alpha	1.10 ± 0.14	2.14 ± 0.35 **
		beta	0.71 ± 0.07	1.02 ± 0.06 **
	blue	delta	2.43 ± 0.23	3.19 ± 0.02 ***
		theta	0.92 ± 0.05	1.30 ± 0.08 ***
		alpha	1.34 ± 0.14	1.80 ± 0.23
		beta	0.71 ± 0.10	0.76 ± 0.07

*p*-values: * < 0.05, ** < 0.001, *** < 0.0001.

## Data Availability

The presented data are available upon reasonable request from the corresponding author.

## Supplementary Materials

The following supporting information can be downloaded at: https://www.mdpi.com/article/10.3390/molecules27134059/s1, Table S1: Amplitude; Table S2: Power.

## Sample Availability

Samples of the compounds are not available from the authors.
